# DNA Extraction and Amplification from Contemporary Polynesian Bark-Cloth

**DOI:** 10.1371/journal.pone.0056549

**Published:** 2013-02-21

**Authors:** Ximena Moncada, Claudia Payacán, Francisco Arriaza, Sergio Lobos, Daniela Seelenfreund, Andrea Seelenfreund

**Affiliations:** 1 Centro de Estudios Avanzados en Zonas Áridas (CEAZA), La Serena, Chile; 2 Departamento de Bioquímica y Biología Molecular, Facultad de Ciencias Químicas y Farmacéuticas, Universidad de Chile, Santiago, Chile; 3 Escuela de Antropología, Área de Ciencias Sociales, Universidad Academia de Humanismo Cristiano, Santiago, Chile; Natural History Museum of Denmark, Denmark

## Abstract

**Background:**

Paper mulberry has been used for thousands of years in Asia and Oceania for making paper and bark-cloth, respectively. Museums around the world hold valuable collections of Polynesian bark-cloth. Genetic analysis of the plant fibers from which the textiles were made may answer a number of questions of interest related to provenance, authenticity or species used in the manufacture of these textiles. Recovery of nucleic acids from paper mulberry bark-cloth has not been reported before.

**Methodology:**

We describe a simple method for the extraction of PCR-amplifiable DNA from small samples of contemporary Polynesian bark-cloth (tapa) using two types of nuclear markers. We report the amplification of about 300 bp sequences of the ITS1 region and of a microsatellite marker.

**Conclusions:**

Sufficient DNA was retrieved from all bark-cloth samples to permit successful PCR amplification. This method shows a means of obtaining useful genetic information from modern bark-cloth samples and opens perspectives for the analyses of small fragments derived from ethnographic materials.

## Introduction

*Broussonetia papyrifera* L (Vent) family Moraceae (paper mulberry) grows naturally in East Asia (China, Korea, Taiwan, Japan) and Mainland Southeast Asia [1]. This tree species is one of the earliest plants to have been cultivated in SE Asia as a major raw material for making textiles and paper [2]. It is a widespread crop species in the Pacific islands introduced by Austronesian speaking peoples [1], [3], [4], as suggested by linguistic evidence [5]. *B. papyrifera* was one of the most important cultivated plants in both Near and Remote Oceania, associated with many economic, political and ritual uses, as still evident today on some islands, particularly in Western Polynesia. The use of plant fibers for the manufacture of textiles has been documented archaeologically for the Upper Paleolithic on the Eurasian continent and particularly the making of bark-cloth in many parts of the world precedes the use of looms. The ancient practice of making bark-cloth and bark paper likely began in Asia, where other species of *Broussonetia* are also known. In China its main use was for the manufacture of paper. From here it spread to Japan in AD 610 and developed into an industry of high quality paper products [6].

### Distribution and Dispersal of *B. papyrifera*

The native distribution of the plant is Eastern Asia, China, Taiwan, Japan and Korea, where it grows in forest margins and mixed deciduous evergreen woodlands up to 2300 meters above sea level [6]. It is a tree that can reach up to 20 meters in height, although in the Pacific it rarely grows above 3–5 meters and is kept as a shrub.

Paper mulberry was taken purposely to all islands in Remote Oceania by Polynesian settlers during the colonization voyages, as far as Hawaii, New Zealand and Easter Island, since it was a basic and necessary item for cultural reproduction. Oral tradition of Easter Island mentions paper mulberry as part of the basic supplies that were taken along [7]. We must assume that it was an important item in Pacific cultures to explain the vast dispersal of the plant to all islands where people settled. Today the plant is still grown on many Polynesian islands, but has disappeared on several, particularly in East Polynesia [4].

The present study is part of a larger project which sets forth to contribute to our understanding of the complex human history in the Pacific, using paper mulberry as a proxy of human migration. In an earlier paper [4] we discussed the recent extinction of the plant on several islands, which limits our understanding of its dispersal history and genetic diversity. A potential approach to surmount the absence of extant plants is to explore the possibility of extracting DNA from the finished products made out of paper mulberry bark. Therefore a key question that arises is if DNA survives the manufacturing process of bark-cloth production; hence the problem of DNA survival is not simply of academic interest, but has practical benefits, as in many situations researchers may not have access to fresh materials [8].

### Bark-cloth (Tapa) in the Pacific

In the Pacific some other trees are also used for making bark-cloth (breadfruit, hibiscus and several fig species), yet in Polynesia, *B. papyrifera* reigns supreme. It has been known for almost 1,500 years as a plant whose bark can be used to make textiles (bark-cloth), commonly named tapa, of various grades up to the highest quality. The name tapa, currently used for generically designating bark-cloth made out of paper mulberry, came into use during the late 19^th^ century, and was originally a Samoan word referring only to the borders of non-decorated cloth. Tapa is prized and valuable, since it is warm, long lasting, soft, flexible and white. In the past, tapa was used for the elaboration of cloaks, skirts, loin clothes and ritual gifts [9], [10], [11]. The making of bark-cloth, its use and role in past Pacific culture cannot be underemphasized.

For the manufacture of bark-cloth, the inner bast of the bark is used. The bast has to be separated from the stem and the outer bark. Clean dry strips of the inner bark are stored rolled up, inner side out, for later use. Once cleaned and scraped the inner bark is soaked in water or seawater to soften it, as in the paper making process. Then the strips of clean and wet bark are beaten on a flat stone or wooden surface with hardwood mallets until they are at least twice the original width. The more the cloth is beaten, the finer it becomes. Sometimes the strips are left to ferment in water before a second beating. Larger strips can be made by overlapping strips of tapa and beating them together [6]. Until relatively recent times tapa was the main source of clothing on many Polynesian islands, until substituted by industrial textiles. Despite this, tapa still holds cultural importance, particularly in Western Polynesia and is having an important cultural comeback in Hawaii. Even today in Samoa, Tonga and other islands tapa is still used for religious purposes and as a symbol of wealth. Tapa is also used in the exchange of gifts and today, although many of the traditional forms of making textiles have been abandoned, their symbolic associations have often been transferred to new industrial fibers. Often as not, traditional and modern textiles share ritual and economic spaces (see for example [12], [13]).

There are artifacts made from tapa in different collections around the world, and some of these were collected at the time of first European contacts, which may be amenable to genetic testing. Each of these artifacts is typically unique to particular cultures or geographic regions and can provide insights into the cultural practices of the people who made them. Since bark-cloth is elaborated from biological material, molecular analyses have the potential to contribute significantly to the study of such artifacts. Molecular genetic markers have numerous potential applications if DNA can be isolated from “difficult” biological material, such as processed and unprocessed wood [14] or feathers [8]. For example, DNA may yield relevant information for identification of endangered tree species [15], authentication of wood remains and control of wood trade [16]. Other authors [17] reported the successful amplification of DNA from waterlogged archaeological oak wood from a marine environment. Likewise, Hartnup *et al.* [18] were able to determine the geographic provenance of Maori cloak feathers from over a hundred different cloaks. Their data suggest that the eastern region of the North Island of New Zealand was the origin of most feathers from the particular cloak analysed. Similar molecular approaches have the potential to discover a wealth of lost information from cultural artifacts worldwide [18].

Diverse molecular markers and fingerprinting techniques are rapidly developing for analyses of plant species, and DNA analysis of bark-cloth specimens could be a useful tool in the identification of un-provenanced specimens. In turn, the use of molecular markers could also be useful to analyse historical bark-cloth artifacts of known provenance from locations where the plant is now extinct to address different questions, such as those related to the dispersal of *B. papyrifera* in the Pacific. Alternatively, molecular markers can reveal the plant species used in the making of the cloth, particularly if different species or a mixture of plants were used in the manufacturing process of these textiles. The basic requirement for this kind of analysis is the isolation of DNA from bark-cloth, and the second requirement is the amplification of this DNA with markers which are informative for the identification of origin of the bark-cloth material. In this paper we report the successful isolation and amplification of DNA from diverse sources of bark-cloth from Remote Oceania.

## Materials and Methods

### Ethics Statement

No specific permits were required for the described field studies. We used fresh plant material that came from the private gardens from two of the authors (AS and DS). The bark-cloth material from Easter Island, Hawaii and Marquesas were gifts to AS. The bark-cloth samples from Samoa and Tonga were bought at the local markets. No material was taken from protected land or National Parks. No endangered or protected species were used.

### Plant and Bark-cloth Samples

**Figure 1:**
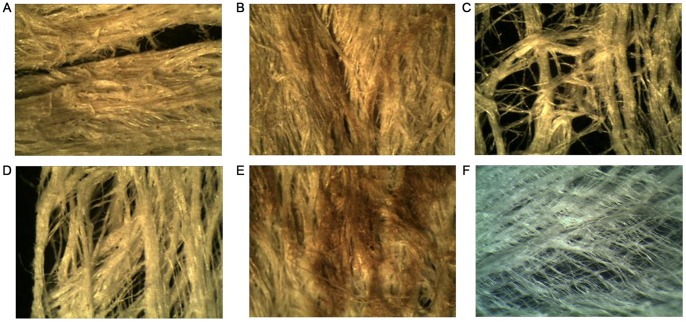
Fiber morphology of contemporary bark-cloth samples from different Polynesian islands. Bark-cloth samples were viewed directly under 10×magnifications. A.-Bark-cloth from Easter Island, Sample 1, B.- Bark-cloth from Easter Island, Sample 2, C.- Bark-cloth from the Marquesas archipelago, D.- Bark-cloth from Samoa, E.- Bark-cloth from Tonga, F.- Bark-cloth from Hawaii.

Six samples of contemporary tapa from five islands were chosen for analysis (Figure 1). We have no particular information or details of the manufacturing process for the samples from Easter Island, Marquesas, Tonga and Samoa. However, we do know that the Hawaiian bark-cloth sample was soaked in seawater for about 3 weeks prior to beating.

Samples of fresh leaves, roots and unbeaten inner bark of an individual paper mulberry plant from Easter Island were included for comparative purposes. Leaves from *Morus alba* of unknown origin sampled in Santiago, Chile, were used as control. In order to prevent contamination, DNA extractions of bark-cloth samples were performed in a physically separated laboratory not dedicated to research of plant samples. All PCR reactions were set up in a UV-treated PCR cabinet.

### Analysis of Fiber Morphology

Fibres were observed under a common binocular lens under 10×magnification and photographed with a digital Tucsen camera 3.0 C (Tucsen Imaging Technology Ltd.).

### Preparation of Plant Material and DNA Extraction

Fragments ranging from approximately 3 mg to 62 mg (of approximately 1 cm^2^), depending of the origin of the bark-cloth sample, were taken from each specimen. Each bark-cloth sample was torn and cut lengthwise with a scalpel on a clean dry glass surface before adding reagents for DNA extraction. Samples of fresh unbeaten inner bark were obtained by peeling young stems and processed with a scalpel as described for bark-cloth. Root samples were finely sliced with a scalpel. Fresh leaves were ground in a small mortar using a porcelain pestle at room temperature.

### DNA Extraction

DNA extractions of bark-cloth samples were performed with and without pretreatment with *Trichoderma viride* cellulase (Cat. N° 150584, from MP BioMedicals LLC, Solon, Ohio, USA) prepared at 5 mg/ml in sodium acetate 3 M pH 5.0. Treatment consisted in incubation of samples at 40°C for 0, 1, 3, 5 hours or overnight. DNA was extracted from all samples based on the protocol described by Lodhi *et al.* [19]. Briefly, this protocol consisted of a homogenization of the plant tissue in an extraction buffer containing 2% CTAB, incubation at 60°C, extraction of proteins and impurities with organic solvents (chloroform and isoamyl alcohol) and precipitation of nucleic acids using cold absolute ethanol in the presence of salt. Finally, DNA was treated with RNase and resuspended in distilled water. Modifications of this protocol consisted in avoiding the use of PVP and increasing the β-mercaptoethanol concentration of the extraction buffer to 1%. For each extraction set, negative controls were included which were performed simultaneously. These negative controls contained extraction buffer but lacked the bark-cloth sample and were treated exactly as all samples for the rest of the extraction protocol. Bark-cloth samples were not treated with RNase, as degradation of RNA was assumed. Each set of DNA extractions from bark-cloth was performed on different days. DNA concentration and quality (Absorbance ratio 260 nm/280 nm) were measured using a NanoDrop ND-2000 spectrophotometer (NanoDrop Technologies, Wilmington, DE, USA) according to the user’s manual.

### PCR Amplification

PCR amplification was performed to examine the success of DNA extraction from bark-cloth and plant tissues. Two different kinds of molecular markers, the ITS region and a nuclear microsatellite marker were chosen. In each case, PCR reactions were carried out using 20–30 ng of DNA from *Morus* leaf samples, 350–550 ng of DNA from *B. papyrifera* leaves, 30–100 ng of DNA from fresh bark and root samples and 20–900 ng of DNA from bark-cloth samples. All PCR reactions included positive reaction controls (DNA extracted from fresh *Morus* and *B. papyrifera* leaves) and a negative PCR reaction control (H_2_O).

### PCR Amplification Using ITS Markers

The region comprising genes 18S and 26S was amplified by PCR using ITS-4 (5′-GCTTAAACTCAGCGGGTAGC-3′) and ITS-5B (5′-TCGCGAGAAGTCCACTGAA-3′) primers (as kindly suggested by Dr. K.-F. Chung, National Taiwan University, personal communication). The ITS1 region was amplified using primers ITS-A (5′-GGAAGGAGAAGTCGTAACAAGG-3′) and ITS-C (5′-GCAATTCACACCAAGTATCGC-3′) [20]. PCR-reaction mixtures consisted of 2.5 mM MgCl_2_, 0.625 mM dNTPs, 0.5 mM of each primer and 0.2 U/µl of GoTaq^R^ Flexi DNA Polymerase (Promega, Madison, WI, USA) in a final volume of 20 µl. Blank reactions were performed by adding the appropriate amounts of sterile distilled water to the reaction in all experiments.

The amplification program for both ITS regions consisted of an initial denaturation step at 94°C during 5 min, followed by 32 cycles (18S to 26S region) or 35 cycles (ITS1 region) with a denaturation step at 94°C for 1 min, an annealing stage at 60°C for 1 min, an extension at 72°C for 1 min and a final extension at 72°C for 7 min. Amplicons were analysed by electrophoresis on 1.5% agarose gels, dyed with GelRed Nucleic Acid Gel Stain (Biotium, Inc.) and visualized under UV light. At least 2 independent reactions were performed for each sample.

### Sequence Analysis

Samples were purified using the DNA Clean and Concentrator Kit™ from Zymo Research, Irvine, CA, USA) according to the manufacturer’s instructions and sequenced at Macrogen Inc. (Seoul, South Korea). For bioinformatic analysis, polymorphisms were visualized and checked on electropherograms from all sequences using Bio Edit 7.1.3.0 software [21]. ITS sequences were edited manually with the test version of EditSeq (DNAstar Lasergene v7.1.0) and aligned using the Clustal W method with MegAlign from the same software [22], [23].

### PCR Amplification of Microsatellite Marker

Microsatellite sequences (SSR) were amplified using primers of locus SS05 Fw (5′-TCCAGCAAAGATGTGACAAAAGTT-3′) and SS05 Rv (5′-TTGCCTTCCCGATTATGCTG-3′) designed originally for *Morus* species [24]. PCR-reaction mixtures consisted of 2.5 mM MgCl_2_, 0.625 mM dNTPs, 0.20 mM of each primer and 0.2 U/µl of GoTaq^R^ Flexi DNA Polymerase (Promega, Madison, WI, USA) in a final volume of 20 µl. Blank reactions were performed by adding the appropriate amounts of sterile distilled water to the reaction in all experiments. The amplification program consisted of an initial denaturation step of 94°C during 5 min, followed by 35 cycles with a denaturation step at 94°C for 1 min, an annealing stage of 57°C for 1.5 min, an extension step at 72°C for 1.5 min and a final extension at 72°C for 5 min. Amplicons were analyzed by electrophoresis on 1.5% agarose gels, dyed with GelRed Nucleic Acid Gel Stain (Biotium, Inc.) and visualized under UV light. At least 2 independent reactions were performed for each bark-cloth sample. As controls, DNA samples from leaves from *M. alba* and leaves, root and bark tissues from *B. papyrifera* were included.

## Results

### Fiber Morphology

As shown in figure 1 the samples vary in coarseness, density and colour. Bark-cloth samples from Easter Island were dense, coarse and of beige or brown colour. The bast fibers are bound closely together. The bark-cloth samples from the Marquesas archipelago and Samoa were of a thin, porous texture and a creamy white colour. In contrast, the sample from Tonga was relatively dense and presented brown spots over a beige background colour, possibly due to the application of dyes. The finest bark-cloth was from Hawaii, which was flexible and very thin, of paper-like quality and very white. It contained fewer impurities, possibly due to a more complex process of preparation involving several cycles of soaking and beating.

### DNA Extraction

At least three DNA extractions were performed from each sample of bark-cloth using cellulase pretreatments of 0, 1 and 3 hours. Incubations of 5 hours and overnight were discarded as they did not yield amplifiable products and did not seem to be useful in increasing DNA extraction performance (data not shown). Contrary to expectations, cellulase treatment for 3 or more hours did not increase DNA yields; it is not clear if this was due to the effect of a prolonged incubation at 60°C or to the plausible presence of traces of contaminant enzymes from the cellulase purified from *T. viride*. In the case of Hawaiian bark-cloth, only one DNA extraction was performed in the absence of cellulase treatment.

**Figure 2:**
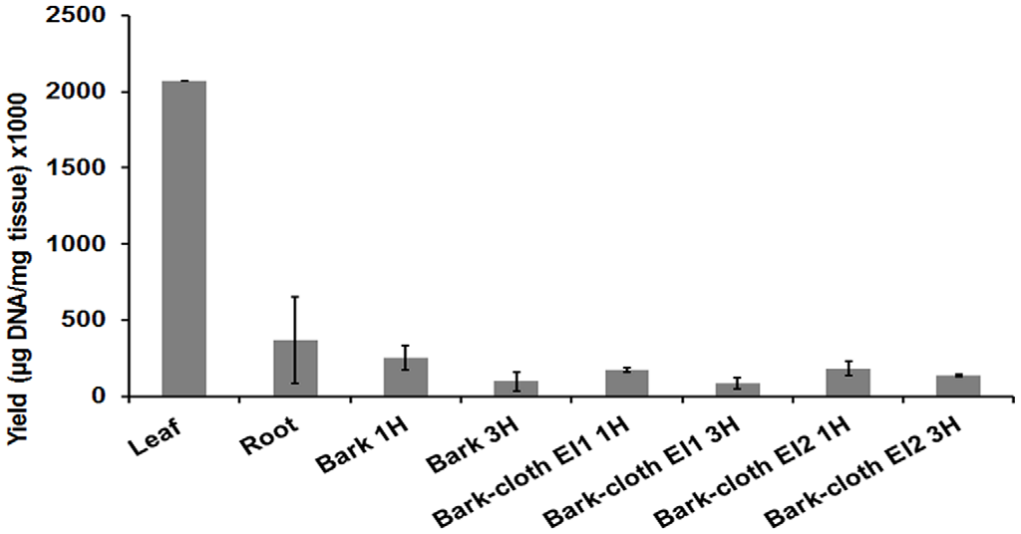
Comparison of DNA yield extracted from fresh tissues from an individual *B. papyrifera* plant and from bark-cloth from Easter Island. Yields obtained from DNA extractions of leaves, root and fresh bark. 1 H: One hour treatment with cellulase; 3 H: 3 hours treatment with cellulase; EI1: Easter Island sample 1; EI2: Easter Island sample 2. Standard deviations from triplicates are indicated.

For comparative purposes, DNA extraction of leaves, roots and fresh bark tissue were also performed from a single individual from Easter Island. The yield and properties of the DNA obtained from these tissues and from bark-cloth samples are summarized in Figure 2, which shows that the yield of DNA extracted from leaves (2.07 µg DNA/mg tissue) was about one order of magnitude larger than that obtained from the other plant tissues, whereas the yield of DNA from bark-cloth (0.08 to 0.18 µg DNA/mg tissue) was similar to that from bark tissue (0.10 to 0.25 µg DNA/mg tissue), according to spectrophotometric measurements.

**Figure 3:**
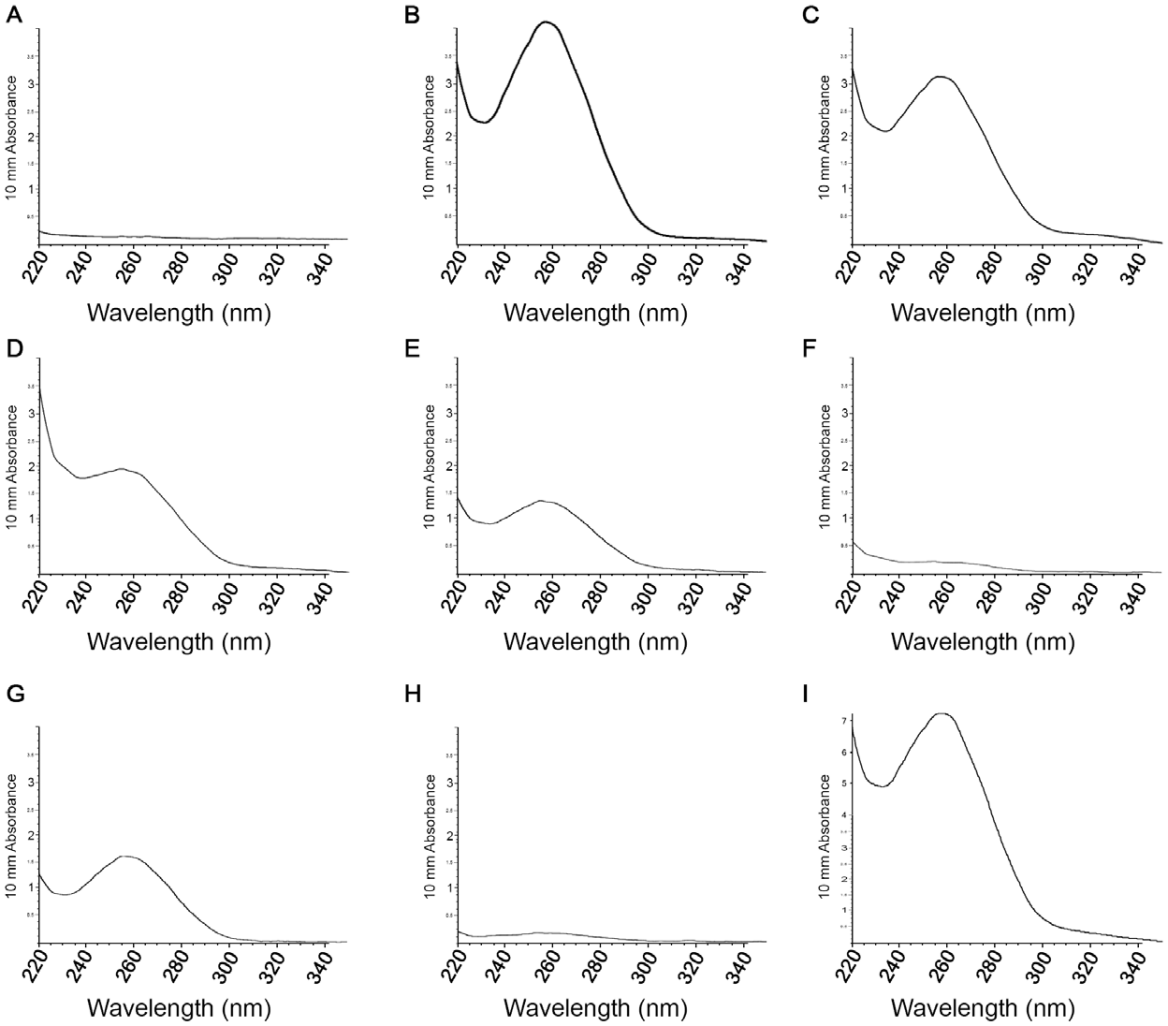
Absorption profiles of DNA extracted from fresh *B. papyrifera* tissues and bark-cloth samples. A: Negative control; B: *B. papyrifera* (leaf); C: *B. papyrifera* (bark); D – I: *B. papyrifera* bark-cloth from D: Easter Island, sample 1; E: Easter Island, sample 2; F: Marquesas; G: Samoa; H: Hawaii; I: Tonga.

Table 1 summarizes the general properties of the analyzed bark-cloth samples. Given the differences in density and homogeneity of the textile fibers, a range of sample weights were obtained for sample sizes of about 1 cm^2^. Average sample weight ranged from 8.6 mg to 27.7 mg (Marquesas and Hawaii) to the thicker and coarser samples from Easter Island which weighted between 15.9 to 41.7 mg. The spectrophotometric absorption profiles for each DNA extraction showed a maximum at 260 nm for all samples. A representative profile for each kind of sample is presented (Figure 3). The negative control did not display absorption at any wavelength (Figure 3A), while DNA profiles from *B. papyrifera* leaf and bark (Figures 3B and 3C, respectively), which were both extracted from fresh tissue, displayed the expected maximum absorption at 260 nm. The DNA extracted from bark-cloth samples also showed the characteristic absorption spectrum. Average DNA concentration values ranged from 8.0 to 17.3 ng/µl in the case of the Marquesan bark-cloth, to 125.7 to 314.1 ng/µl from the Tongan bark-cloth sample, which yielded the maximum DNA amount (see Table 1). Negative controls showed average DNA concentration values that are similar to background levels (0.7–4.6 ng/µl). The ratio of 260/280 values corresponded to pure DNA samples, except in the case of Tongan bark-cloth, which presented a minimum ratio of 1.39 and Easter Island 1 bark-cloth, which presented a maximum ratio of 2.55. Values below 1.8 can be explained by the presence of contaminants [25], whereas a ratio higher than 2.0 nm indicates that the samples could be contaminated with residual chloroform from the extraction procedure [26]. It is difficult to ascribe ratios over 2.0 to the presence of RNA, as has been previously described [25], since is not very plausible that RNA is still present, especially as single stranded nucleic acid molecules, considering that the bark is subjected to a prolonged and complex treatment during the making of bark-cloth. Bark-cloth DNA extracts from Easter Island sample 1 and the Tongan sample were purified using the Genomic DNA Clean & Concentrator ™ Kit (Zymo Research), however this procedure did not improve DNA concentration, nor the 260/280 absorbance ratio or PCR performance, and therefore was omitted in the remaining samples.

**Table 1:** Details of the bark-cloth samples used in the study.

Geographic Origin ofBark-cloth Sample	Cellulase treatmenthours (replicates)	Sample weight (mg)	DNA Concentration(ng/µl)	Range A_260_/A_280_	Yield (µgDNA/mgbark-cloth)	Amplifiable ITS-1 DNA	Amplifiable SSR DNA locus SS05
Easter Island 1	0 H (3)	15.9±2.2	28.4±14.6	1.76–2.55	0.170±0.07	yes	no
	1 H (3)	31.9±19.5	54.9±32.7	1.85–2.00	0.169±0.02		
	3 H (3)	41.7±19.5	28.2±19.6	1.87–2.00	0.082±0.04		
Easter Island 2	0 H (3)	19.0±6.7	65.0±28.6	1.89–2.16	0.340±0.10	yes	yes
	1 H (3)	23.8±7.9	39.2±5.7	1.90–1.97	0.180±0.05		
	3 H (3)	22.3±6.2	49.4±31.0	1.61–1.93	0.211±0.11		
Marquesas	0 H (3)	11.0±5.8	17.3±10.7	1.90–1.98	0.155±0.03	yes	yes
	1 H (3)	8.6±2.6	8.5±3.3	1.76–1.97	0.096±0.01		
	3 H (3)	11.2±2.2	8.0±1.6	1.85–1.89	0.072±0.01		
Samoa	0 H (3)	12.2±4.0	70.9±11.8	1.97–2.10	0.655±0.27	yes	no
	1 H (3)	16.2±2.4	58.4±14.3	1.96–2.03	0.357±0.05		
	3 H (3)	16.5±2.4	32.0±4.7	1.83–1.91	0.196±0.02		
Tonga	0 H (3)	23.9±1.2	314.1±47.2	1.82–2.05	1.306±0.15	yes	yes
	1 H (3)	26.9±2.0	211.8±107.3	1.39–1.87	0.813±0.47		
	3 H (3)	28.1±3.8	125.7±30.1	1.49–1.75	0.458±0.12		
Hawaii	0 H (1)	27.7	7.7	1.88	0.028	yes	Not tested
Negative control	0 H (4)	–	4.6±3.9	1.61–2.25	-	no	no
	1 H (3)	–	1.0±0.8	0.88–2.95	-		
	3 H (2)	–	0.7±0.7	0.47–3.27	-		

In an attempt to obtain DNA from bark-cloth samples using a non-invasive treatment, we also incubated the same samples overnight in extraction buffer, under three different conditions: 1) 16 hours at room temperature, 2) 16 hours at 60°C and 3) one hour incubation at 60°C followed by 16 hours incubation at room temperature. Samples were then submitted to the extraction protocol described above. These extraction products were also analyzed spectrophotometrically and some samples exhibited a characteristic DNA profile with absorption maxima at 260 nm (data not shown). However, none of these extraction procedures yielded amplifiable DNA using ITS and SS05 microsatellite markers, with either GoTaq DNA Polymerase (Promega) or Herculase Enhanced DNA Polymerase (Agilent Technologies) using the PCR protocol recommended by the supplier.

### ITS Amplification and Sequencing

**Figure 4:**
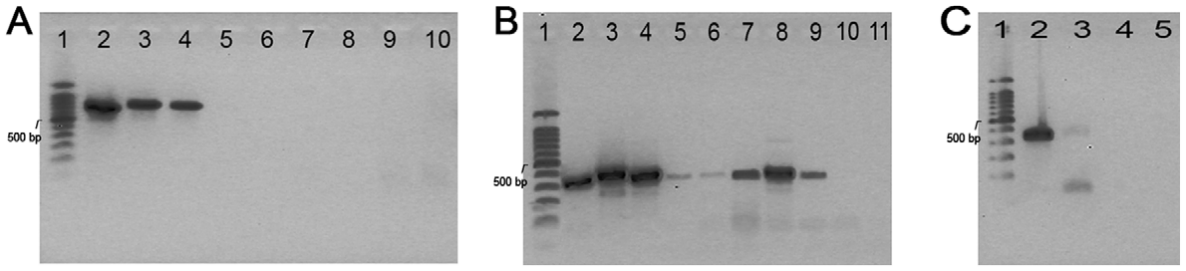
Amplification of the ITS region using DNA obtained from bark-cloth. A.- Complete ITS region, lane 1∶100 bp DNA Ladder, lane 2: *Morus* (leaf), lane 3: *B. papyrifera* 1 (leaf), lane 4: *B. papyrifera* 2 (leaf), lanes 5 to 9: bark-cloth samples. Lane 5: Easter Island 1, lane 6: Easter Island 2, lane 7: Tonga, lane 8: Marquesas, lane 9: Samoa, lane 10: H_2_O. B.- ITS1 region, lane 1∶100 bp DNA Ladder, lane 2: *Morus* (leaf), lane 3: *B. papyrifera* 1 (leaf), lane 4: *B. papyrifera* 2 (leaf), lanes 5 to 9: bark-cloth samples. Lane 5: Easter Island 1, lane 6: Easter Island 2, lane 7: Tonga, lane 8: Marquesas, lane 9: Samoa, lane 10: Negative control, lane 11: H_2_O. C.- ITS1 region, lane 1∶100 bp DNA Ladder, lane 2: *Morus* (leaf), lane 3: bark-cloth from Hawaii, lane 4: Negative control, lane 5: H_2_O.

DNA extracts from bark-cloth were assayed for amplification of the complete ITS region, spanning the ITS1-5, 8S-ITS2 sequences, that in *B. papyrifera* are of approximately 700 bp [27]. The same samples were also subjected to amplification of the ITS1 region of about 300 bp. Results showed that attempts to amplify the complete ITS region were unsuccessful (Figure 4A), confirming that DNA from bark-cloth has suffered degradation during the manufacturing process. Interestingly, expected amplicons of 300 bp were obtained for ITS1 from all bark-cloth samples tested (Figure 4B and 4C).

The ITS1 sequence information for all bark-cloth samples indicated that they corresponded to *B. papyrifera* material of Polynesian origin (GenBank accession number HM623778.1), as they displayed the characteristic polymorphism found in samples originating from this region of the Pacific [27]. Negative controls and/or blank controls (water) did not show presence of DNA (Figure 4A, lane 10, Figure 4B, lanes 10 and 11 and Figure 4C, lanes 4 and 5).

### Microsatellite Markers

**Figure 5:**
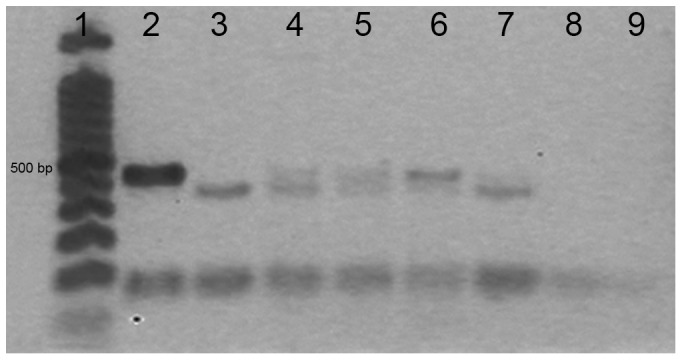
Amplification of the microsatellite SS05 locus using DNA obtained from different *B. papyrifera* tissues and bark-cloth. Lane 1∶100 bp DNA Ladder, lane 2: *Morus* (leaf), lane 3: *B. papyrifera* (leaf), lane 4: *B. papyrifera* (fresh bark), lane 5: *B. papyrifera* (root), lane 6: *B. papyrifera* (root hair), lane 7: Easter Island bark-cloth sample 2, lane 8: Negative control, lane 9: H_2_O.

As at present there are no published SSR loci for *B. papyrifera*, we performed amplifications with a microsatellite marker developed for the related genus *Morus*. We assayed locus SS05, which displays an allele size range of 342–478 bp in mulberry [24]. The chosen marker exhibited transferability to *Broussonetia*, as DNA from different *B. papyrifera* tissues could be amplified showing a band or double-band of similar size than *Morus* (Figure 5, lanes 3 to 6). In the case of DNA extracts from bark-cloth positive PCR amplification was obtained, as shown by Easter Island sample 2 in Figure 5, lane 7. Negative and blank controls (water) did not show evidence of DNA (Figure 5, lanes 8 and 9). Successful amplification was achieved for three (Easter Island 2, Marquesas and Tonga) of five bark-cloth samples assayed, as summarized in Table 1.

## Discussion

In this study we used samples of bark-cloth from different origins and textures to establish a protocol to extract amplifiable DNA. As shown in Figure 1, the different fibres were quite dissimilar in coarseness, colour and density. Nonetheless, it was possible to extract DNA from all tested samples. For achieving this, pieces of bark-cloth were subjected to a simple protocol based on mechanical shearing or grinding of the material and the use of CTAB detergent. Compared to yields obtained from fresh leaves, DNA extracted from bark-cloth was significantly lower (Figure 2).

DNA extractions from bark-cloth showed spectrophotometric absorption profiles with a characteristic maximum at 260 nm and most samples exhibited an appropriate 260/280 ratio. Although purity ratios and spectral profiles are important indicators of sample quality, the best indicator of DNA or RNA quality is functionality in the downstream application of interest [19]; in our case, PCR amplification for molecular marker analysis. We first chose to analyse the ITS region as this is a well-known robust marker and because its sequence corresponds to a multicopy region of the nuclear genome. We evaluated the amplification of the complete ITS region, a sequence of approximately 700 bp, which amplified leaf DNA, but failed in all bark-cloth samples (Figure 4A). In contrast, the ITS1 region, which spans only 300 pb was successfully amplified in the same samples (Figure 4B). These results are probably due to the fragmentation of nucleic acids that occurred during the manufacturing of bark-cloth. Breakdown of genomic DNA has been described in ancient plant material, suggesting that fragments of more than 300 bp often cannot be amplified from extracts of ancient plant DNA [28]. Due to its harsh treatment during manufacture, bark-cloth may be considered equivalent to ancient plant material.

We then assayed the amplification of the microsatellite locus SS05 (originally developed for *Morus*) on the same DNA samples obtained from bark-cloth. Amplification products of approximately 400 bp in three out of five bark-cloth samples were obtained. In comparison to ITS sequences which correspond to multicopy regions, microsatellites are single copy nuclear markers, a fact that may explain the lack of amplification of these sequences in some samples due to mechanical shearing. This result suggests that bark-cloth specimens from different islands may have been subjected to dissimilar treatments during manufacture or storage, and some might have been more aggressive on DNA integrity. No direct correlation between texture and success in PCR amplification could be observed, based on the relative coarseness of the bark-cloth samples, as both the thick coarse cloth from Easter Island and the thin almost white sample from the Marquesan archipelago yielded amplification products.

The bark-cloth samples used for this study are all contemporary material, and not museum specimens or ancient material. We used these modern samples as proof of principle for the extraction of DNA and to implement an appropriate protocol based on molecular markers for later application to historic materials. It is expected that only small amplification products can be obtained, therefore the target regions for PCR reactions should be between 80 and 300 bp [28], and previous knowledge of the target DNA sequences chosen for analysis is necessary. Also, markers which are present in multiple copies such as chloroplast or nuclear ribosomal (ITS) markers have more chances of survival than single copy markers. This is in agreement with reports of DNA survival in other materials, such as leather, where multicopy mitochondrial DNA survives the tanning process, whereas single copy nuclear markers do not [29]. As with ancient plant DNA, when applying these protocols to museum specimens, precautions will have to be taken to avoid contamination with modern plant DNA.

In our case, cross contamination between bark-cloth and fresh tissues from the same species was avoided by using a separate laboratory to perform DNA extractions, and amplification reactions were then set up in a UV-treated PCR cabinet. DNA amplifications were achieved only for fragment lengths of around 300 bp (ITS1 and SSR); sequencing of ITS1 fragments revealed the presence of DNA ascribed to the Polynesian haplotype of *B. papyrifera* [27]. Moreover, our negative and blank controls were shown to be free of contaminant DNA in PCR reactions for both ITS and SSR markers.

As museums are wary of removal of even small pieces of material from intact historical or ethnographic bark-cloth specimens for destructive analyses, it is necessary to find small detached pieces from specimens in order to perform this kind of study. The small size of fragments used in this work (1 cm^2^ or less) may satisfy the requirements of museum curators. As discussed above, the use of molecular markers for the analysis of cultural materials can provide valuable insights into the cultural practices of the people who made them. Since bark-cloth in Polynesia is made mainly but not exclusively from *B. papyrifera* fibres, molecular markers could also be a useful tool for identifying the botanical species used to manufacture bark-cloth. Molecular markers may also proof helpful for the identification of bark-cloth specimens of unknown provenance. A wealth of historical information housed in international collections can therefore be explored with minimal damage to valuable ethnographic artifacts.

Non-invasive extraction procedures are extremely valuable for the analysis of museum samples, as shown for insect collections [30] and would have been of immense value for examining intact historical tapa specimens. The possibility of using non-invasive methods opens unprecedented possibilities of research at a molecular level; unfortunately a non-invasive procedure assayed in this study did not yield positive results.

Contemporary, historic and ancient plant materials have been studied using appropriate molecular markers, as reported for processed, submerged and unprocessed wood [14]–[17] and also for papyrus [31]. This study on bark-cloth adds a new source of materials of plant origin for genetic analysis. Overall, this work outlines a simple method showing that it is possible to extract and amplify DNA from bark-cloth using molecular markers for the analysis of ethnographic material. The present protocol may not be limited to paper mulberry bark-cloth, and can prove useful on other kinds of cultural materials made of plant fibres. The feasibility of genetic analysis opens up new scenarios for the study of textile materials of cultural importance.

## References

[1] Matthews P (1996) Ethnobotany, and the origins of *Broussonetia papyrifera* in Polynesia: an essay on tapa prehistory. In: Davidson JM, Irwin G, Leach BF, Pawley A, Brown D, editors. Oceanic Culture History: Essays in Honour of Roger Green. New Zealand Journal of Archaeology Special Publication. 117–132.

[2] Howard M (editor) (2006) Bark-cloth of Southeast Asia. Studies in the Material Cultures of Southeast Asia 10. Bangkok: White Lotus Press. 309 p.

[3] Prebble M (2008) No fruit on that beautiful shore: What plants were introduced to the subtropical Polynesian islands prior to European contact? In: Clark G, Leach F, O’Connor S, editors. Islands of Enquiry: Colonisation, seafaring and the archaeology of maritime landscapes. Terra Australis 29. ANU E-Press. 227–251.

[4] Seelenfreund D, Clarke AC, Oyanedel N, Piña R, Lobos S, et al (2010) Paper mulberry (*Broussonetia papyrifera*) as a commensal model for human mobility in Oceania: anthropological, botanical and genetic considerations, New Zealand J Bot 48. 3: 1–17.

[5] Green RC (1979) Early Lapita art from Polynesia and Island Melanesia: Continuities in ceramic, barkcloth, and tattoo decorations. In: Mead SM, editor. Exploring the Visual Art of Oceania: Australia, Melanesia, Micronesia and Polynesia. Honolulu, University Press of Hawaii. 13–31.

[6] Barker C (2002) Plant portraits: 432. *Broussonetia papyrifera*, Moraceae. Curtis’s Bot Magazine 19 (1): 8–18.

[7] Métraux A (1971) Ethnology of Easter Island. Bernice Bishop Museum Bulletin 160. Honolulu, Hawaii. Bishop Museum Press Reprints. 432 p.

[8] Olsen ME, Friis Bengtsson C, Bertelsen MF, Willerslev E, Gilbert MTP (2012) DNA from keratinous tissue Part II: Feather. Annals of Anatomy 194: 31–35. 21489767 10.1016/j.aanat.2011.03.003

[9] Kennedy R (1934) Bark-cloth in Indonesia. JPS 43: 229–43.

[10] Kooijman S (1988) Polynesian Barkcloth. Shire Ethnography, UK. Shire Publications Ltd. 64 p.

[11] Bell LA (1988) Papyrus, Tapa, Amate and Rice Paper: paper making in Africa and the Pacific, Latin America and Southeast Asia. McMinniville, Oregon, Liliaceae Press. 464 p.

[12] Colchester C (2003) Clothing the Pacific. Oxford & New York, Berg Publishers. 215 p.

[13] Küchler S, Were G (2005) The Art of Clothing: A Pacific Experience. Cavendish Publishing UK. UCL Press. 171 p.

[14] Rachmayanti Y, Leinemann L, Gailing O, Finkeldey R (2009) DNA from processed and unprocessed wood: Factors influencing the isolation success. Forensic Sci Int: Genetics 3: 185–192. 19414167 10.1016/j.fsigen.2009.01.002

[15] Asif MJ, Cannon CH (2005) DNA extraction from processed wood: a case study for the identification of an endangered timber species (*Gonystylus bancanus*). Plant Mol Biol Rep 23: 185–192.

[16] Deguilloux M-F, Pemonge M-H, Petit RJ (2002) Novel perspectives in wood certification and forensics: dry wood as a source of DNA. Proc Roy Soc London B 269: 1039–1046. 10.1098/rspb.2002.1982 PMC1690996 12028761

[17] Speirs A, McConnachie G, Lowe A (2009) Chloroplast DNA from 16th century waterlogged oak in a marine environment: initial steps in sourcing the Mary Rose timbers. In: Haslam M, Robertson G, Crowther A, Nugent S, Kirkwood K, editors. Archaeological science under a microscope: studies in residue and ancient DNA analysis in honour of Thomas H. Loy. Terra Australis 30. ANU E-Press. 175–189.

[18] Hartnup K, Huynen L, Te Kanawa R, Shepherd LD, Millar CD, et al (2011) Ancient DNA recovers the origins of Māori feather cloaks. Mol Biol Evol 28(10): 2741–2750. 21558445 10.1093/molbev/msr107

[19] Lodhi MA, Guang-Ning Y, Norman FW, Bruce IR (1994) A simple and efficient method for DNA extraction from grapevine cultivars and *Vitis* species. Plant Mol Biol Rep 12: 6–13.

[20] Blattner FR (1999) Direct amplification of the entire ITS region from poorly preserved plant material using recombinant PCR. BioTechniques 27: 1180–1186. 10631497 10.2144/99276st04

[21] Hall TA (1999) BioEdit: a user-friendly biological sequence alignment editor and analysis program for Windows 95/98/NT. Nucl Acids Symp Series 4: 95–98.

[22] Thompson JD, Higgins DG, Gibson TJ (1994) CLUSTAL W: improving the sensitivity of progressive multiple sequence alignment through sequence weighting, position-specific gap penalties and weight matrix. Nucl Acids Res 22: 4673–4680. 7984417 10.1093/nar/22.22.4673 PMC308517

[23] Higgins D, Sharp PM (1988) CLUSTAL: a package for performing multiple sequence alignments on a microcomputer. Gene 73: 337–244. 3243435 10.1016/0378-1119(88)90330-7

[24] Zhao W, Miao X, Jia S, Pan Y, Huang Y (2005) Isolation and characterization of microsatellite loci from the mulberry, *Morus L.* Plant Sci. 168: 519–525.

[25] Varma A, Padh H, Shrivastava N (2007) Plant genomic DNA isolation: An art or a science. Biotechnol J 2: 386–392. 17285676 10.1002/biot.200600195

[26] Borges DB, Amorim MB, Waldschmidt AM, Mariano-Neto E, Vivas CV, et al (2012) Optimization of DNA extraction from fresh leaf tissues of *Melanoxylon brauna* (Fabaceae), Genet Mol Res. 11: 1586–1591. 10.4238/2012.May.22.8 22653632

[27] Seelenfreund D, Piña R, Ho K-Y, Lobos S, Moncada X, et al (2011) Molecular analysis of *Broussonetia papyrifera* (L.) Vent. (Magnoliophyta: Urticales) from the Pacific, based on ribosomal sequences of nuclear DNA. New Zealand J Bot 49: 413–420.

[28] Schlumbaum A, Tensen M, Jaenicke-Després V (2008) Ancient plant DNA in archaeobotany. Veget Hist Archaeobot 17: 233–244.

[29] Vuissoz A, Worobey M, Odegaard N, Bunce M, Machado CA, et al (2007) The survival of PCR-amplifiable DNA in cow leather. J Archaeol Sci 34 (5): 823–829.

[30] Gilbert MT, Moore W, Melchior L, Worobey M (2007) DNA extracting from dry museum beetles without conferring external morphological damage. PlosOne 2: e272. 10.1371/journal.pone.0000272 PMC1803022 17342206

[31] Marota I, Basile C, Ubaldi M, Rollo F (2002) DNA decay rate in papyri and human remains from Egyptian archaeological sites. Am J Phys Anthropol 117: 310–318. 11920366 10.1002/ajpa.10045

